# Assessing accuracy and consistency in intracranial aneurysm sizing: human expertise vs. artificial intelligence

**DOI:** 10.1038/s41598-024-65825-4

**Published:** 2024-07-12

**Authors:** Andrej Planinc, Nina Špegel, Zala Podobnik, Uroš Šinigoj, Petra Skubic, June Ho Choi, Wonhyoung Park, Tina Robič, Nika Tabor, Leon Jarabek, Žiga Špiclin, Žiga Bizjak

**Affiliations:** 1 Medilab Diagnostic Imaging, Vodovodna 100, 1000 Ljubljana, Slovenia; 2 Faculty of Medicine, University of Ljubljana, Ljubljana, Slovenia; 3 Department of Neurological Surgery, Asan Medical Center, University of Ulsan College of Medicine, Seoul, Republic of Korea; 4 Laboratory of Imaging Technologies, Faculty of Electrical Engineering, University of Ljubljana, Ljubljana, Slovenia

**Keywords:** Risk factors, Biomedical engineering

## Abstract

Intracranial aneurysms (IAs) are a common vascular pathology and are associated with a risk of rupture, which is often fatal. Aneurysm growth of more than 1 mm is considered a surrogate of rupture risk, therefore, this study presents a comprehensive analysis of intracranial aneurysm measurements utilizing a dataset comprising 358 IA from 248 computed tomography angiography (CTA) scans measured by four junior raters and one senior rater. The study explores the variability in sizing assessments by employing both human raters and an Artificial Intelligence (AI) system. Our findings reveal substantial inter- and intra-rater variability among junior raters, contrasting with the lower intra-rater variability observed in the senior rater. Standard deviations of all raters were above the threshold for IA growth (1 mm). Additionally, the study identifies a systemic bias, indicating a tendency for human experts to measure aneurysms smaller than the AI system. Our findings emphasize the challenges in human assessment while also showcasing the capacity of AI technology to improve the precision and reliability of intracranial aneurysm assessments, especially beneficial for junior raters. The potential of AI was particularly evident in the task of monitoring IA at various intervals, where the AI-based approach surpassed junior raters and achieved performance comparable to senior raters.

## Introduction

Intra- and inter-rater differences in intracranial aneurysm (IA) size measurement can affect their radiological characterization^1–3^ and upon based treatment decision. Clinical practice is generally inclined towards preventive endovascular or neurosurgical IA treatment, but these options come with a high risk of procedure-related complications^4^. Hence, small IAs frequently go untreated in patients because the risk of complications from treatment is thought to be higher than the risk of rupture. The majority of patients with small IAs undergo frequent computed tomography or magnetic resonance angiographies (CTA and MRA, resp.) to monitor the development of IA over time. If a size increase (i.e. growth) of an IA is observed, it is usually prioritized for treatment because growing IAs are more likely to rupture than the stable ones^5–9^.

Growth of an aneurysm is determined by assessing its size from consecutive angiographic scans. The primary parameter for measurement is the aneurysm’s dome diameter. The determination of whether an aneurysm has undergone growth relies on the disparity between the measurements performed in baseline and follow-up angiographic scans. If the IA size measurements increase for at least 1 mm in any direction^10^, the aneurysm is categorized as having undergone growth and is therefore associated with an elevated risk of rupture. However, the established size increase threshold for identifying aneurysm growth is positioned well within the range of potential intra- and inter-rater variability, therefore determining the IA growth through manual size measurement seems rather unreliable.

In practice, the clinicians typically use manual annotation tools and a 2D slice-by-slice viewer to select two dome wall endpoints and thereby measure the IA size on the three-dimensional (3D) MRA and CTA scans, or if in the interventional suite, on 3D digital subtraction angiography (DSA) or even two-dimensional (2D) DSA. The use of manual 2D measurements has inherent limitations, such as limited perspectives of the aneurysm that might not accurately represent the entirety of its dimensions^11,12^. The variability in the 2D measurement plane selection further contributes to the variability of size measurement, in addition of the variability of endpoint selection, both within the same and among different raters^13,14^.

In contemporary research, numerous engineering research teams have introduced computational methodologies and software solutions to offer a more objective, precise, and consistent assessment of the IA morphology^15,16^. Nevertheless, in clinical practice, the healthcare professionals continue to rely predominantly on the manual 2D size measurements. This prevalent reliance on traditional techniques might stem from the lack of (i) comprehensive comparative analysis between the manual measurements and those obtained using these emerging computerized tools, and (ii) experimental evidence of enhancing the accuracy and/or consistency in morphological evaluation, and (iii) their alignment with the prevailing norms and practices in the clinical domain.

This study focuses on the accuracy and reproducibility aspects of IA size measurement with three key objectives: (1) quantification of intra- and inter-rater variability of IA size measurements of one senior and four junior raters, (2) comparison of the accuracy and precision of computerized 3D versus the manual 2D IA size measurements, and (3) validation of the proficiency of junior versus senior raters’, and versus the computerized 3D measurement approach for assessing aneurysm growth. The implications of this study’s findings highlight the potential of computerized 3D IA size measurements to provide a valuable assistance to clinicians, enhancing the standardization of aneurysm morphology evaluation.

## Experiments and results

This study assesses and compares IA size measurements obtained from various human raters, using 2D manual size assessment, and a computerized 3D measurement. Human raters that participated in the assessment of IA size were young doctors or medical students in their final years (junior raters 1–4) and one neuroradiologist with > 15 years of experience in the field (senior rater).

**Figure 1:**
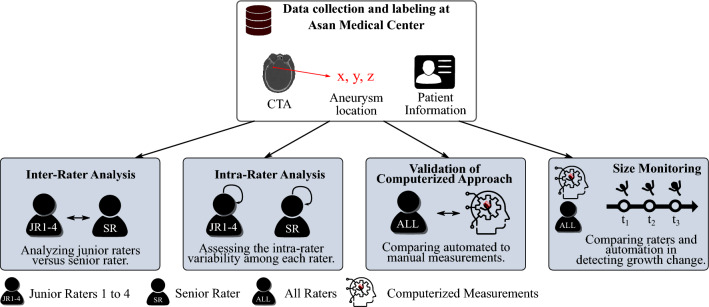
Summary of the study’s four experiments: inter-rater analysis, intra-rater analysis, validation of a computerized approach, and size monitoring.

Each junior rater independently performed 484 IA size measurements on 248 CTA images, measuring 126 IAs twice. The senior rater conducted 246 IA measurements, including 63 instances where the IAs were measured twice. The CTA data were anonymized and presented to the raters in randomized order; hence, during the experiment the raters were not aware they were performing repeated measurements. The computerized 3D IA measurements were obtained for all IA. For more details regarding the dataset, refer to Sect. 0.1.

The study encompassed four experiments that evaluated (1) intra-rater variability, (2) inter-rater variability, (3) a comparative analysis between the computerized and manual measurements, and (4) impact of the measurement values on the assessment of IA growth status (see Fig. 1).

### Inter-rater analysis

**Figure 2:**
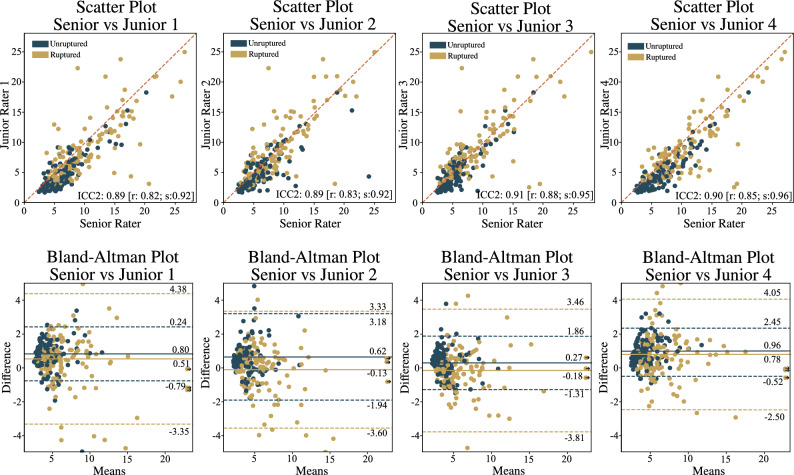
Inter-rater variability analysis of IA size measurement for each of four junior raters versus the senior rater, with corresponding scatter and Bland-Altman plots (*top* and *bottom rows*, respectively).

The first set of experiments compared the performance of junior raters versus the senior rater. Figure 2 shows the scatter diagrams and reports the pairwise intra-class correlation coefficient (ICC) values, separately for the ruptured and unruptured IAs. Similarly, the Bland-Altman plots report corresponding systematic (average difference) and random errors (standard deviation).

The average differences between junior raters and the senior rater in the manual IA size measurements were smaller for ruptured cases compared to stable cases (0.51 vs 0.8, − 0.13 vs 0.62, − 0.18 vs 0.27, 0.78 vs 0.96, respectively). Conversely, the standard deviations of the measurements were generally higher for the ruptured cases than for the stable ones. Figure 2 illustrates that the variability of the manual measurements was generally higher for the larger ruptured IAs but not for the unruptured ones.

We conducted comparisons between all raters by computing Cohen’s d effect size for the IA size measurements across all combinations of raters (see Table 1). Comparing senior rater to junior raters 1, 2, 3 and 4 we obtained Cohen’s values 0.18, 0.07, 0.02 and 0.25, respectively. The measurements for junior raters 1 and 4 had somewhat larger discrepancies to the senior rater’s measurements, while the measurements by junior raters 2 and 3 were well aligned with senior’s ones.

When comparing the measurements between the junior raters we observed that the comparison between junior rater 1 and 4, as well as between junior rater 2 and 3, yielded a rather low Cohen’s d value of 0.06. Other combinations of junior raters yielded Cohen’s d values between 0.12 and 0.24 (see Table 1).

**Table 1:** Inter-rater Cohen’s d effect size analysis.

Cohen’s D	Junior 1	Junior2	Junior 3	Junior 4	Senior
Junior 1	0.0	0.12	0.17	0.06	0.18
Junior 2	0.12	0.0	0.06	0.19	0.07
Junior 3	0.17	0.06	0.0	0.24	0.02
Junior 4	0.06	0.19	0.24	0.0	0.25
Senior	0.18	0.07	0.02	0.25	0.0

The ICC2 analysis, as outlined by Koo et al.^17^, for inter-rater variability indicates *good agreement* (0.75–0.90) or *excellent agreement* (above 0.9) between junior raters and the senior rater (0.89, 0.89, 0.91, and 0.91 for raters 1–4, respectively). The ICC2 analysis based on the rupture vs. unruptured IA groups reveals *excellent agreement* for all the raters in the unruptured group (0.92, 0.92, 0.95, and 0.96 for raters 1–4, respectively) and *good agreement* for the ruptured group (0.82, 0.83, 0.88, 0.85 for raters 1–4, respectively). Note that throughout this paper, we adhere to the interpretation guidelines for the ICC2 values as provided by Koo et al.^17^.

### Intra-rater analysis

For the intra-rater analysis, each junior rater conducted repeated measurements on a set of 126 intracranial aneurysms (IAs), while the senior rater undertook the same for 63 IAs. To prevent measurement memorization effects, cases were anonymized using random names. The order of assessment was randomized in the first and second assessment round, performed with a three weeks break.

**Figure 3:**
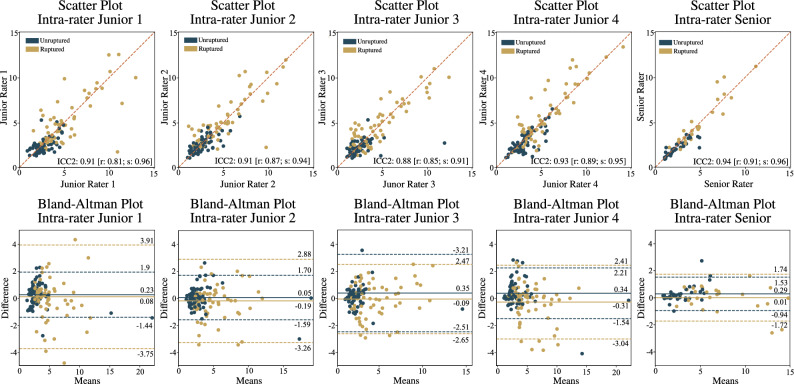
Intra-rater variability analysis of IA size measurement for each of four junior raters and the senior rater, with corresponding scatter and Bland-Altman plots (*top* and *bottom rows*, respectively).

The intra-rater variability analysis is provided through scatter and Bland-Altman plots for each rater shown in Fig. 3. Junior rater 1 exhibited an average intra-rater difference of 0.23 mm and 0.08 mm, with standard deviations of 1.67 mm and 3.8 mm for unruptured and ruptured cases, respectively. Junior rater 2 demonstrated mean differences of 0.05 mm and − 0.19 mm, with standard deviations of 1.65 mm and 3.07 mm for unruptured and ruptured groups. Junior rater 3 showed mean differences of 0.35 mm and − 0.09 mm, with standard deviations of 2.68 mm and 5.12 mm for unruptured and ruptured groups, respectively. Junior rater 4 achieved differences of 0.34 mm and − 0.31 mm, with standard deviations of 1.87 mm and 2.72 mm for unruptured and ruptured groups, respectively. The senior rater exhibited differences of 0.29 mm and 0.01 mm, with standard deviations of 1.23 mm and 1.73 mm for unruptured and ruptured groups, respectively.

The intra-rater Cohen’s d analysis revealed low values for all raters (0.06, 0.03, 0.07, 0.01, and 0.07 for Junior raters 1, 2, 3, 4, and the senior rater, respectively). The analysis indicated generally smaller Cohen’s d values for the stable group (0.01, 0.07, 0.04, 0.02, and 0.01 for Junior raters 1, 2, 3, 4, and the senior rater, respectively) compared to the ruptured group (0.17, 0.03, 0.10, 0.02, and 0.03 for Junior raters 1, 2, 3, 4, and the senior rater, respectively).

The intra-rater ICC2 variability analysis demonstrates good agreement (0.75–0.90) or excellent agreement (above 0.9) for all raters (0.91, 0.91, 0.88, and 0.93 for junior raters 1–4, respectively, and 0.94 for the senior rater). The ICC2 analysis, based on the rupture vs. unruptured group, indicates excellent correlation for all junior raters in the unruptured group (0.96, 0.94, 0.91, and 0.95 for junior raters 1–4, respectively) and good correlation for the ruptured group (0.81, 0.87, 0.85, 0.89 for junior raters 1–4, respectively). The senior rater achieved excellent correlation for both groups (0.96 for the unruptured group and 0.91 for the ruptured group).

### Validation of computerized measurements

**Figure 4:**
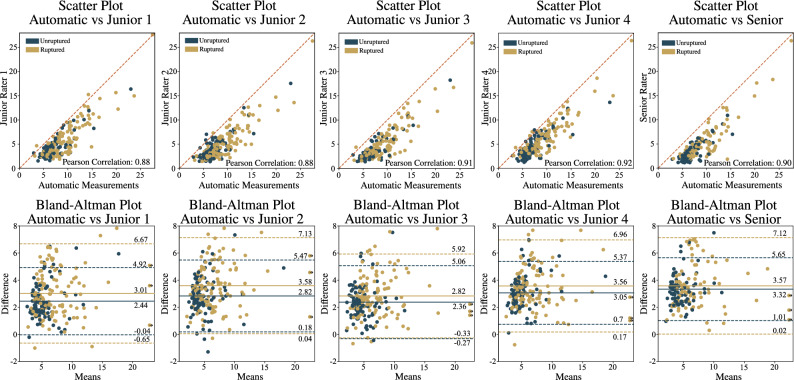
Analysis of manual IA size measurement versus automated measurements for all raters, with corresponding scatter and Bland-Altman plots (*top* and *bottom rows*, respectively).

The automated measurements were compared to manual measurements, and the agreement and consistency can be observed from the scatter and Bland-Altman plots in Fig. 4 for each rater versus the automated approach. The results indicated that junior raters 1, 2, 3, and 4 had mean differences of 2.44 mm, 2.82 mm, 2.36 mm, and 3.05 mm for the unruptured group and 3.01 mm, 3.58 mm, 2.82 mm, and 3.56 mm for the ruptured group, respectively, when compared to the computerized measurements. The corresponding standard deviations were 2.48 mm, 2.64 mm, 2.66 mm, and 2.33 mm for the unruptured group and 3.66 mm, 3.5 mm, 3.12 mm, and 3.39 mm for the ruptured group, respectively. For the senior rater, mean differences of 3.32 mm and 3.57 mm were achieved for the unruptured and ruptured groups, respectively, with standard deviations of 2.32 mm and 3.55 mm for the unruptured and ruptured groups, respectively.

**Figure 5:**
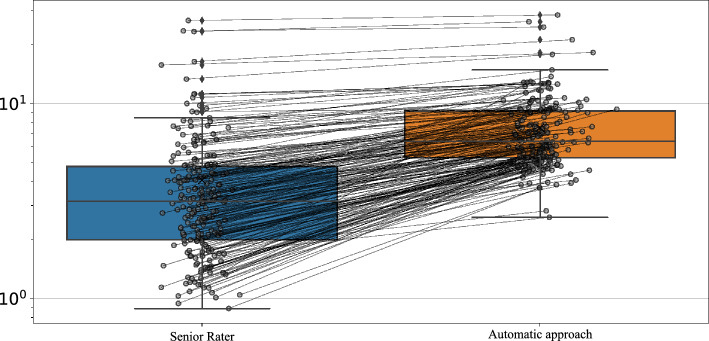
Distribution of the senior rater’s and computerized measurements. Corresponding measurements are indicated by the *connecting lines*.

To further evaluate the mean disparity between measurements conducted by the senior rater and computerized measurements, we display box-and-whisker plots in Fig. 5 that illustrate the distribution of these measurements and associate the corresponding measurements via connecting lines. The consistent inclination of the connecting lines on Fig. 5 implies a variance between the computerized measurements and those conducted by the senior rater. This corroborates the findings from the Bland-Altman plots (see Fig. 4), indicating a systematic bias between the senior rater and computerized measurements (3.32 and 3.57 for unruptured and ruptured aneurysms, respectively).

The agreement and consistency between the computerized measurements and the manual measurements was assessed using the ICC. The senior rater demonstrated a robust ICC of 0.90, while the junior raters achieved comparable ICC values of 0.92, 0.88, 0.91, and 0.88, respectively.

### Monitoring size over time

**Figure 6:**
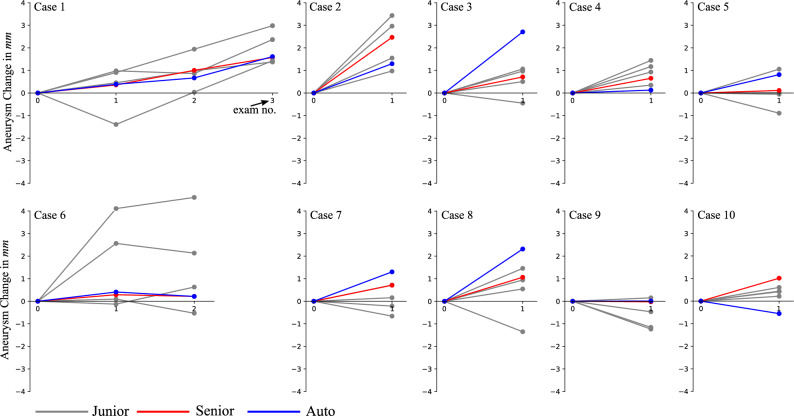
Aneurysm size measurements over time for the junior raters (*gray lines*), senior rater (*red lines*), and the computerized approach (*blue lines*).

For a small group of 10 patients in the dataset, who had been imaged at multiple time-points, we have analyzed the performed manual and computerized measurements over time with the aim to establish the validity of size measurements to identify IA’s morphological status (e.g. growing, stable or shrinking). Figure 6 depicts the obtained size measurements for the 10 cases. With the exception of cases 3 and 10, the disparity between the measurements between the senior and the junior raters is smaller than the SD of the senior rater’s measurements. Notably, the measurements provided by junior raters exhibit more substantial deviations from the senior rater’s measurements in contrast to the computerized measurements.

## Discussion

In current clinical practice, the risk of rupture for IAs is closely linked to factors like aneurysm size, its growth rate, and prior instances of subarachnoid hemorrhage (SAH), among others. Well-known predictive models for rupture, such as ELAPSS^18^ and PHASES^10^, primarily rely on the size of the IA. Additionally, when surgery is not recommended, regular monitoring of IA shape is advised. If an intracranial aneurysm grows more than 1 mm^10^, it is generally classified as a growing intracranial aneurysm. It is established that growing IA are more likely to rupture, meaning measurements of baseline and follow-up aneurysms need to be precise and repeatable. This underscores the need for precise and repeatable measurements of baseline and follow-up aneurysm sizes.

However, manual measurements of intracranial aneurysms can vary due to factors like image quality, imaging modality, the experience of the rater, and external elements such as fatigue, night-time conditions, and heavy workload^19^. Furthermore, the variability in measurements arising from manual annotation that involves subjective judgement, for instance in 2D viewing plane selection and endpoint localization, can either obfuscate the actual changes in size in growing IAs, or lead to false growth indications in stable IAs. In this study we conducted an analysis of the intra-rater variability involving four junior raters and one senior rater.

The examination of inter-rater variability through Bland-Altman plots in Fig. 2, comparing junior raters to the senior rater, revealed that all junior raters exhibited a low mean value (average 0.66 [0.27–0.96] for the nonruptured group and average 0.25 [-0.18–0.78] for the ruptured group). The standard deviation ranged from 0.5 to 2.56 mm for the nonruptured group and from 3.27 to 3.63 mm for the ruptured group. D’Argento et al.^20^, who also explored inter-rater variability among experienced raters, reported a corresponding standard deviation value of approximately 1.5 mm. This discrepancy is anticipated given the difference in the raters’ experience levels. The agreement among the five raters was assessed through Cohen’s d effect size. According to Cohen’s original work^21^, effect sizes of 0.2 are considered small, 0.5 medium, and 0.8 large; however, these benchmarks are somewhat arbitrary and should not be strictly adhered to^22^. In the comparison of raters (see Table 1), the observed effect sizes ranged from 0.06 to 0.24, signifying small effect sizes. This indicates that there is no substantial difference between the raters.

The junior raters exhibited rather large standard deviations in their measurements (avg. 1.96 [1.65–2.68] for unruptured and 3.67 [2.72–5.12] for ruptured group), while the senior rater displayed a substantially smaller standard deviation of 1.23 mm for unruptured and 1.73 for ruptured group. The nearly doubled standard deviation observed in the ruptured group of junior raters compared to senior raters can be attributed to the challenge of distinguishing the aneurysm’s edge from the adjacent blood pool, introducing ambiguity into the measurements. While this discrepancy is expected due to differences in experience between the junior and senior raters, the results underscore that the intra-rater variability for all raters exceeds the threshold set for determining aneurysm growth.

An alternative to manual measurement is the use of deep learning-based segmentation and measurement for IA. The computerized measurement process for IA size involved four main steps: vascular segmentation, selection of the region of interest, isolation of the IA, and measurement of IA size by determining the largest diameter of the isolated dome. By comparing our automatic measurements to manual measurements conducted by a senior rater (who demonstrated an intra-rater standard deviation of 1.23 mm), we observe a standard deviation of 2.32 mm for the unruptured group and 3.55 mm for the ruptured group. Mean differences are 3.32 and 3.57 for the ruptured and unruptured groups, respectively. This pattern mirrors findings from a previous study by Rajabzadeh-Oghaz et al.^23^, where a mean difference of 2.01 mm was noted between computerized and corresponding manual measurements. This variability may be attributed to the fact that computerized measurements were conducted in 3D space, whereas manual measurements relied on a selected 2D viewing plane, likely introducing inconsistencies.

While we recognize that the standard deviation of automatic artificial intelligence-based algorithms is not yet at the level of a senior rater, it underscores an area that requires improvement. However, the algorithm excels in monitoring changes in IA size, where the emphasis is on aneurysm change rather than standard deviation. We assume that when assessing IA growth, the intelligence-based algorithms will consistently produce a similar deviation at each time point. This consistency can be attributed to the similar IA morphology observed across all time points. To evaluate and compare artificial intelligence-based measurements with human raters, we conducted measurements on a group of 10 patients who underwent angiographic scanning at multiple time points. This examination aimed to assess the level of agreement among junior raters, senior raters, and computerized measurements, with the senior rater’s measurements serving as the established benchmark. Visual observations revealed considerable variability in the measurements of junior raters. In Fig. 6, six instances (cases 1, 2, 5, 6, 8, 9) showed inconsistent IA morphology changes compared to the senior rater. Conversely, computerized measurements were inconsistent in only one, possibly two cases. Specifically, in case 3, computerized measurements indicated growth, while the senior rater classified the aneurysm as stable. In case 10, both the senior rater and the computerized method characterized the aneurysm as stable. However, the computerized measurement indicated a slight decrease in IA size, while the senior rater’s measurements indicated a minor increase. Thus, the assessment of IA change using artificial intelligence-based measurements not only proves robust and repeatable but also surpasses the performance of junior raters, underscoring its superior reliability.

Accurate assessment of intracranial aneurysm growth, often defined by a mere 1 mm change in aneurysm size^18^, is pivotal. The intricacies of intra- and inter-rater variability, however, can introduce the potential for wrong indication, falsely categorizing an aneurysm’s status as growing, stable or shrinking in follow-up images. Even though the senior raters exhibit lower variability, it remains slightly above the 1 mm threshold for growth indication. Such erroneous determinations hold the grave consequences of both surgical complications and spontaneous rupture. Addressing these concerns, a computerized aneurysm size measurement approach emerges as a reliable and consistent tool for quantifying intracranial aneurysm morphology change. This approach not only aids in precise measurements but also offers an invaluable second opinion. Particularly beneficial for junior raters, computerized approaches like the one proposed can significantly enhance the evaluation of intracranial aneurysms. Furthermore, by embracing computerized measurements, the monitoring of intracranial growth can be effectively shielded from the influences of intra- and inter-rater variability, thus ushering in a new era of reliability and accuracy in clinical decision-making.

## Methods

### Data

We collected a set of 248 CTA scans from the ASAN medical center, Seoul, Republic of Korea, all of which featured at least one untreated IA. Among these scans, there were 145 male and 90 female patients, with a median age of 57 for males and 51 for females. Among 235 patients assessed, 8 received a single follow-up scan, while one patient underwent two follow-up scans, and another underwent three. The average duration between successive scans was 1340 days, with a range from 547 to 2738 days. These 10 patients underwent follow-up scans to monitor the potential growth of aneurysms throughout their lives.

The CTA acquisitions were conducted on 11 CT scanners, specifically the models GE LightSpeed Pro 16, GE LightSpeed QX/i, GE Optima CT660, GE LightSpeed VCT, GE Discovery CT750 HD, GE HiSpeed CT/I, GE Optima CT660, and GE Revolution CT.

A proficient radiologist with over 15 years of experience, who was not involved as a rater in this study, meticulously analyzed the CTA scans. The analysis involved locating the aneurysms within the scans and determining whether they had previously ruptured based on the patient’s clinical records. The study identified a total of 358 IAs, among which 201 had experienced rupture, while 157 were unruptured. The observed IAs had a median diameter of 3.38 mm, with 52% categorized as small (diameter $<3$ mm), 35% as medium-sized (3 mm < diameter $<7$ mm), and 13% as large-sized (diameter $>7$ mm). Aneurysm sizes were categorized as small, medium, or large using measurements from clinical center reports. None of our raters were involved in generating these original reports, ensuring unbiased assessment.

Importantly, the ruptured IAs present in the dataset were all imaged within a week after the rupture event, and previous research had indicated minimal morphological changes in the aneurysm shape prior to rupture, as compared to their appearance after rupture^24^. Notably, previously treated aneurysms were not included in this study even though they were present in the dataset.

The study encompassed aneurysms from five distinct anatomical locations. The largest subset of IAs was located at the middle cerebral artery bifurcation, totaling 119 cases. Additionally, there were 69 aneurysms on the anterior communicating artery, 5 on the posterior communicating artery, and 53 on the anterior choroidal artery. In the posterior circulation, 39 cases were identified, while the anterior cerebral artery featured 21 cases. Further details regarding the dataset can be found in Table 2.

**Table 2:** Dataset information.

Patient and case information		Anatomical location of IA	
Patients (male/female)	235 (90/145)	Middle cerebral artery bifurcation	119 (33%)
Median patient age (male/female)	54 (57/51)	Anterior communication artery	69 (19%)
No. of CTA scans	248	Posterior communication artery	57 (16%)
No. of IAs (unruptured/ruptured)	358 (157/201)	Anterior choroidal artery	53 (15%)
IA size, small$^{\star }$/medium$^{\dagger }$/large$^{\ddagger }$	186/125/47	Posterior circulation	39 (11%)
No. of patients with 1/2/3 follow-up scan	8/1/1	Anterior cerebral artery	21 (6%)

$^{\star }$Diameter $<3$ mm; $^{\dagger }$diameter $>3$ and $<7$ mm); $^{\ddagger }$diameter $>7$ mm.

### Human raters

In this investigation, measurements were conducted by a team consisting of four junior raters and one senior rater. The junior raters, comprising young doctors or students in the final years of their medical studies, lacked prior specialized training in IA measurements. This scenario simulated the conditions typically encountered by radiologists at the outset of their specialization, as formal training for IA size measurement is generally not provided, based on our experience. Nonetheless, to ensure consistency in this study, our senior rater, possessing over 15 years of experience, conducted an instructional session for the junior raters before commencing with the measurements. The initial segment of the session encompassed a comprehensive overview of general IA morphology, definition of IA neck, potential case variations, fundamental aspects of the CTA modality, and the fundamental principles underlying the adjustment of window and width settings for obtaining accurate measurements. The subsequent phase of training consisted of live demonstrations illustrating the localization of endpoints and the extraction of the IA size measurement, all in diverse measurement scenarios and with the utilization of the designated software^25^. During this phase, the raters were introduced the dataset without revealing any particulars about which cases were repetitions or involved multiple scans from the same patients. Subsequently, all junior raters participated in a practical training session supervised by the senior rater. This hands-on session allowed them to seek clarification by posing questions while conducting the IA size measurements. Moreover, if uncertain, they were given the option to skip a particular aneurysm, and to later engage in a follow-up session with the senior rater for clarification. During this session, the focus was on refining their ability to identify the CTA slice that best visualizes the aneurysm, although the senior rater refrained from assisting in localizing the endpoints needed for the size measurement or confirming measurement accuracy.

### Manual measurement protocol

All CTA imaging data for the cases underwent anonymization and were stored on a single computer equipped with an integrated Digital Imaging and Communications in Medicine (DICOM) viewer^25^ capable of performing the requisite measurements. To ensure any potential bias stemming from the use of different workstations was eliminated, uniformity was maintained as all raters employed the identical setup. The data for each cases encompassed source CTA scan, patient gender and age information, coordinates denoting the global location of the aneurysm, and the aneurysm’s status (ruptured or unruptured). To obviate software-related divergences in image assessment among raters, exclusive utilization of the provided DICOM viewer was mandated.

A standardized measurement protocol was adhered to by each rater. Initially, the DICOM image was loaded into the software using drag-and-drop actions. While raters were instructed to solely employ 1 mm sliced images for performing measurements, the option to examine 5 mm sliced images was available upon preference. Subsequently, the rater input coordinates specifying the global location of the aneurysm, which consequently adjusted the software’s field of view to the approximate aneurysm position. Once the appropriate 3D volume view slice and angle were selected, the rater initiated the measurement of the intracranial aneurysm by holding down the left mouse button, thereby selecting the first endpoint. The second endpoint was recorded when the left mouse button was released. Next to the line connecting the two endpoints, the measured distance between the two was displayed and automatically copied to the clipboard. In the event that a rater deemed the IA measurement acceptable, they proceeded to paste the corresponding value into the Microsoft Excel table of measurements.

### Computerized measurement

The computerized measurement process for IA size involved four main steps: (1) vascular segmentation, (2) selection of the region of interest (ROI), (3) isolation of the IA, and (4) measurement of IA size by determining the largest diameter of the isolated dome. In steps 1 and 3, trained and validated models from prior studies were utilized^26,27^. No further training was conducted specifically for this study. Hence, in this section, we provide a condensed overview of the methodology and findings.

For **vascular segmentation**, we employed a self-adapting framework named nnU-Net^28^. The preprocessing was fully automated following the nnU-Net pipeline. Initially, all cases underwent cropping based on a minimal bounding box encapsulating foreground intensity values (i.e., CTA intensity $>-1024$). This reduction in computational load was achieved without loss of relevant information. The CTA’s intensity values were resampled using third-order spline interpolation, while resampling of associated vessel masks employed nearest neighbor interpolation. Normalization was applied to all non-zero voxels by mean subtraction and division by standard deviation. Given our high-resolution 3D scan datasets, we configured the nnU-Net architecture as “3dfullres” to deploy a 3D U-Net CNN model^29^. In this encoder-decoder architecture, downsampling in the encoder utilized convolution layers with a stride of 2, while upsampling in the decoder employed transposed convolutions with a stride of 2. The nnU-Net framework automatically selected patches of size 96$\times$160$\times$160 and a batch size of 2. The Adam optimizer was employed for 150 epochs with an initial learning rate of $0.9 \times 10^3$. A training and validation dataset of 210 CTA cases from the same dataset were used. The mean Dice similarity coefficient (DSC) was reported and used for further analysis across all test folds (trained using 5 folds), yielding a mean DSC of 0.91 for the nnUnet model trained and tested on the 210 CTAs. Following the nnUnet vessel segmentation, the marching cubes and the smooth non-shrinking algorithms^30,31^ were applied so as to reconstruct corresponding 3D surface mesh of the aneurysm and surrounding vessels.

**Extraction of the ROI** on the vascular mesh surrounding the particular IA was based on the coordinates of each IA’s center, as previously annotated by a radiologist. The nearest point to the global IA coordinates on the vascular segmentation served as the starting point to identify 5000 mesh points closest according to geodesic distance. This strategy ensured the inclusion of proximal vessels connected to the IA, while excluding nearby peripheral vessels. While the selected number of 5000 connected points sufficed for all the aneurysms within this dataset, the number would likely need to be increased in case of giant aneurysms.

**Isolation of the IA** was performed by a geometric classifier learning approach proposed in a prior study^27^. The approach was trained and validated on an expanded dataset, utilizing the publicly available dataset named IntrA^32^ for this purpose. The IntrA consists of 103 3D models of entire brain vessels extracted from the MRA scans, featuring 116 IAs. Surface meshes from the IntrA dataset were directly employed without reprocessing. The procedure for IA detection and isolation was not explicitly detailed in the paper.

For our purposes a skilled neurosurgeon manually isolated the aneurysm from surrounding vessels by delineating a closed curve on each extracted surface mesh. The closed curve was annotated as an ordered sequence of points on the surface mesh, with the last point connecting the first to obtain a closed curve. The line segments connecting the two point in a sequence where projected to the surface mesh according to smallest geodesic distance, thus yielding the final closed curve isolating the mesh points on either side. Those points closest to IA center were labeled as the aneurysm, while the other as vessel points. Using the above procedure the surgeon’s flexibility in annotating the points and the closed curve was not confined to a specific aneurysm neck shape. The obtained manual IA isolations were used for training and validation of a multi-layer neural network (MNN) point classification model named PointNet++^33^. The achieved true positive rate (TPR) for the point classification task on the IntrA dataset was 0.96. The obtained point classication model was then applied to the surface mesh in the CTA-extrated ROI.

**Automated computation of IA size measurement** involved finding the maximum distance (MaxLen) between two points of the isolated IA dome mesh. Notably, the MaxLen cannot be directly compared to rater measurements of IA size due to its consideration of the entire 3D space as opposed to a single 2D slice, potentially leading to systematially larger, but consistent measurements.

### Evaluation metrics

In this study, we employed various data visualization techniques to analyze intra- and inter-rater differences, assess the correlation between raters using the interclass correlation score, and establish trends in measurement comparison through box plots enhanced with visual connections.

A fundamental technique in analytical chemistry and biomedicine, the Bland–Altman plot^34^ or difference plot, was utilized for evaluating the concordance between distinct assays. The Bland-Altman plot enables the identification of potential biases in mean differences and estimation of the range within which 95% of the disparities between the second and first methods reside. Both unit differences and percentage differences plots were employed to scrutinize the data. Importantly, the Bland-Altman plot method solely delineates the extent of agreements and refrains from rendering judgments about the acceptability of these ranges.

To quantify the correlation among all raters, the interclass correlation score (ICC)^35^ was harnessed. We adopted the implementation provided by Pingouin^36^. As we utilized a fixed group of *k* raters assessing each target, the application of ICC to a broader rater population is not applicable. Although we eliminated mean differences among raters, we retained sensitivity to interactions. This customized modification of the ICC score is denoted as ICC2 in Pingouin.

In the context of comparing two measurement groups, a comparative strategy involved plotting two adjacent box plots. Notably, these box plots were augmented with visual connections that provide insights into measurement trends, thereby facilitating the estimation of prevailing tendencies between measurements.Č

## Data Availability

The data used in this study are available upon a reasonable request to the corresponding author.
