# Author Correction: Assessing accuracy and consistency in intracranial aneurysm sizing: human expertise vs. artificial intelligence

**DOI:** 10.1038/s41598-024-69217-6

**Published:** 2024-08-06

**Authors:** Andrej Planinc, Nina Špegel, Zala Podobnik, Uroš Šinigoj, Petra Skubic, June Ho Choi, Wonhyoung Park, Tina Robič, Nika Tabor, Leon Jarabek, Žiga Špiclin, Žiga Bizjak

**Affiliations:** 1 Medilab Diagnostic Imaging, Vodovodna 100, 1000 Ljubljana, Slovenia; 2 Faculty of Medicine, University of Ljubljana, Ljubljana, Slovenia; 3 Department of Neurological Surgery, Asan Medical Center, University of Ulsan College of Medicine, Seoul, Republic of Korea; 4 Laboratory of Imaging Technologies, Faculty of Electrical Engineering, University of Ljubljana, Ljubljana, Slovenia

Correction to: *Scientific Reports* 10.1038/s41598-024-65825-4, published online 12 July 2024

In the original version of this Article Nina Špegel, Zala Podobnik, Uroš Šinigoj, Petra Skubic, Tina Robič & Nika Tabor were incorrectly affiliated with ‘Medilab Diagnostic Imaging, Vodovodna 100, 1000, Ljubljana, Slovenia’. The correct affiliation is listed below.

Faculty of Medicine, University of Ljubljana, Ljubljana, Slovenia

The original Article has been corrected.

